# Lowered Plasma Steady-State Levels of Progesterone Combined With Declining Progesterone Levels During the Luteal Phase Predict Peri-Menstrual Syndrome and Its Major Subdomains

**DOI:** 10.3389/fpsyg.2019.02446

**Published:** 2019-10-30

**Authors:** Chutima Roomruangwong, André F. Carvalho, Frank Comhaire, Michael Maes

**Affiliations:** ^1^Department of Psychiatry, Faculty of Medicine, Chulalongkorn University, Bangkok, Thailand; ^2^Department of Psychiatry, University of Toronto, Toronto, ON, Canada; ^3^Centre for Addiction and Mental Health, Toronto, ON, Canada; ^4^Endocrinology and Metabolic Disease, Ghent University Hospital, Ghent, Belgium; ^5^Fertility Clinic, Aalter, Belgium; ^6^Department of Psychiatry, Medical University Plovdiv, Plovdiv, Bulgaria; ^7^IMPACT Research Center, Deakin University, Geelong, VIC, Australia

**Keywords:** premenstrual syndrome, depression, anxiety, physio-somatic, fatigue, progesterone

## Abstract

**Background:**

It is unknown whether lowered steady state levels of sex hormones coupled with changes in those hormones during the menstrual cycle are associated with premenstrual syndrome (PMS).

**Objective:**

To examine associations between levels of progesterone and oestradiol during the menstrual cycle and PMS considering different diagnostic criteria for PMS.

**Methods:**

Forty-one women aged 18–45 years with a regular menstrual cycle completed the Daily Record of Severity of Problems (DRSP) for all 28 consecutive days of the menstrual cycle. Blood was sampled at days 7, 14, 21, and 28 to assay oestradiol and progesterone.

**Results:**

We developed a new diagnosis of peri-menstrual syndrome, which is characterized by increased DRSP severity in pre and post-menstrual periods and increased scores on the major DRSP dimensions, i.e., depression, physio-somatic symptoms, breast tenderness and appetite, and anxiety. This new diagnosis performed better than classical diagnoses of PMS, including that of the American College of Obstetricians and Gynecologists (ACOG). Lowered steady state levels of progesterone, when averaged over the menstrual cycle, together with declining progesterone levels during the luteal phase predict severity of peri-menstrual symptoms. Steady state levels of oestradiol and declining oestradiol levels during the cycle are also related to DRSP severity although most of these effects appeared to be mediated by progesterone.

**Conclusion:**

A significant increase in menstrual-cycle related symptoms can best be conceptualized as “peri-menstrual syndrome” and may result from insufficient progesterone production (relative corpus luteum insufficiency), which, in part may result from lowered oestradiol production indicating suboptimal pre-ovulatory follicular development.

## Introduction

Premenstrual syndrome (PMS) comprises affective, behavioral, and physical symptoms appearing during the luteal phase of the menstrual cycle and ameliorating after the onset of menses (Deuster et al., 1999; Dickerson et al., 2003). Symptoms of PMS include fatigue, depression, cramps, bloating, anxiety and breast tenderness. A recent meta-analysis shows that the prevalence of PMS is 47.8% (95% CI: 32.6–62.9), with a lower prevalence in France, i.e., 12% (95% CI: 11–13), and a higher prevalence in Iran, namely 98% (95% CI: 97–100). This burdersome condition is commonly observed in adolescent girls and young women with prevalence rates between 58.1 to 92.3% among university students (Acikgoz et al., 2017; Hussein Shehadeh and Hamdan-Mansour, 2018). PMS is associated with substantial functional impairment comparable to that observed in dysthymia (Kues et al., 2016) and may lead to impaired work productivity (Chawla et al., 2002; Halbreich et al., 2003) and interfere with marital relationships (Frank et al., 1993), family/homemaking functions (Kuczmierczyk et al., 1992), hobbies and social activities (Heinemann et al., 2010), thereby decreasing health-related quality of life (Farrokh-Eslamlou et al., 2015). Furthermore, PMS is also an important predictor of perinatal depression (Studd and Nappi, 2012; Buttner et al., 2013; Roomruangwong et al., 2016; Stoner et al., 2017).

The normal menstrual cycle results from an integrated action of the hypothalamic-pituitary-ovary axis and the uterine endometrium. The hypothalamus releases gonadotropin-releasing hormone (GnRH) every 1–1.5 h during the follicular phase and every 2–4 h during the luteal phase. GnRH activates the pituitary gland thereby increasing levels of luteinizing hormone (LH) and follicle stimulating hormone (FSH). LH stimulates theca cells of the ovarian follicles to produce androstenedione, whereas FSH stimulates the synthesis of aromatase, which catalyzes the conversion of androstenedione into oestradiol (Barbieri, 2014). During the follicular phase, oestradiol promotes the proliferation of the uterine endometrium, while during mid-cycle, higher concentrations of oestradiol cause a positive feedback to the hypothalamus, resulting in an increased in GnRH secretion and a LH surge, which initiates “ovulation.” After ovulation, the follicle is transformed into a corpus luteum, a yellow mass of cells which secretes progesterone thereby preparing the endometrium for implantation (so-called a “secretory endometrium”) in case fertilization occurs (Barbieri, 2014).

The cyclical nature of PMS and the absence of symptoms among women who underwent bilateral oophorectomy and women during their anovulatory cycles (Rapkin and Winer, 2009; Rapkin and Akopians, 2012) suggest that PMS is associated with reproductive hormones, including progesterone and oestradiol (Seeman, 1996; Case and Reid, 1998; Tan, 2001; Doyle et al., 2007). There is some evidence that progesterone plays a relevant role in the pathophysiology of PMS as symptoms usually appear during the luteal phase when progesterone is produced and released from the corpus luteum, subsequently decreasing in the late luteal phase. For example, a study among 122 healthy, reproductive age women showed that increased progesterone levels in the luteal phase were accompanied by lowered levels of aggression, irritability and fatigue, and additionally that peak progesterone levels in the luteal phase are inversely associated with the same symptoms (Ziomkiewicz et al., 2012). Nevertheless, studies in large study samples failed to demonstrate any efficacy of progesterone in the treatment of PMS (Freeman et al., 1990; Ford et al., 2012). In addition, evidence indicates that PMS-like symptoms may be introduced or re-introduced during cyclical and continuous progesterone treatment (Baker and O’Brien, 2012). Fewer studies, however, have examined possible associations between oestradiol and PMS, although there is some evidence that combined estrogen-progesterone contraception may have some benefits in the treatment of PMS (Freeman et al., 2012; Lopez et al., 2012; Takeda et al., 2015). Such benefits have not been universally demonstrated across studies (Bakhshani et al., 2013). Fluctuations in oestradiol and progesterone levels during the cycle could be more closely associated with the onset of PMS symptoms than their steady-state levels (Schmidt et al., 2017). Nevertheless, it is still unknown whether PMS, severity of PMS and its relevant symptom factors (e.g., depression versus somatic) are associated with steady state levels coupled with changes in sex hormones during the menstrual cycle.

Hence, the aim of this study was to examine associations between steady state levels of progesterone and oestradiol and changes in both hormones during the menstrual cycle and the presence and severity of PMS. We *a priori* hypothesized that lowered steady levels of progesterone and oestradiol coupled with declining levels of these hormones during the menstrual cycle could be associated with the emergence of PMS symptoms.

## Materials and Methods

### Participants

We recruited 41 participants by word of mouth, 21 women without subjective complaints of PMS and 20 women with subjective complaints of PMS. Participants were staff members or friends and relatives of staff members and women accompanying a patient to the hospital. Inclusion criteria were: 1) women aged 18–45 years; 2) a regular menstrual cycle with cycle length 27–30 days during past year; 3) being able to read and write in Thai; 4) willing to have four blood samples drawn at day 7 (T1), day 14 (T2), day 21 (T3) and day 28 (T4) of the menstrual cycle; and 5) complete the DRPS daily for all consecutive days of the menstrual cycle. Exclusion criteria in both groups were: (1) those with a history of psychiatric illness, including schizophrenia, major depression, bipolar disorder, and obsessive compulsive disorder; (2) those with a history of major medical illness, including diabetes type 1, autoimmune or immune-inflammatory disorders such as inflammatory bowel disease, rheumatoid arthritis, psoriasis and multiple sclerosis; (3) those who are currently pregnant or lactating or using hormonal contraceptive agents; and (4) those who are using any psychotropic medications. The body mass index (BMI) was computed as weight (in kg) divided by height 2 (in meter). The study was approved by the Ethics Committee of the Faculty of Medicine, Chulalongkorn University, Bangkok, Thailand (IRB No.611/60, COA No. 1111/2017). Written informed consent was obtained from all participants prior to the study.

### Measures

#### Clinical Assessments

All participants were requested to complete questionnaires comprising personal information including menstrual history, age, education, height, weight, a history of alcohol or substance use, and diets. The latter lifestyle factor was examined in order to exclude subjects with unusual diets, which could affect the immune system (e.g., anti-inflammatory diets). Participants in both groups were evaluated by the same experienced psychiatrist (CR) before recruitment into the study for their potential diagnosis of PMS and psychiatric and medical exclusion criteria. Severity of PMS was scored using the Daily Record of Severity of Problems (DRSP), which was scored daily during the menstrual cycle by all participants. This scale consists of 21 items + 3 functional impairment items commonly used in the evaluation of PMS (Endicott et al., 2006). The DRSP is a self-report test that scores the “presence” and “severity” of premenstrual symptoms and that can be used to make the DSM-IV diagnosis of premenstrual dysphoric disorder (PMDD) (Biggs and Demuth, 2011).

Table 1 shows two different diagnoses of PMS used in the present study. Firstly, we used the American College of Obstetricians and Gynecologists (ACOG) diagnostic criteria for PMS (American College of Obstetricians and Gynecologists, 2014). The ACOG criteria include one or more affective and physical symptoms during 5 days prior to menses in three menstrual cycles and these symptoms must “be relieved within 4 days after onset of menses without recurrence until at least day 13 of the cycle” (American College of Obstetricians and Gynecologists, 2014). Moreover, the subject must experience identifiable dysfunctions in social, academic, or work performance. The diagnosis of PMS was also made when the total DRSP score ≥ 70 on day −5 to −1 of menses and when there was a difference of at least 30% between premenstrual (day −5 to −1) and postmenstrual (day 6–10) scores (Endicott et al., 2006; Biggs and Demuth, 2011; Qiao et al., 2012).

**TABLE 1:** Definition of four different diagnoses used in the current study to diagnose “premenstrual” syndrome.

**Diagnostic Label**	**Abbreviation**	**Definition**	
Premenstrual syndrome (American college of obstetricians and gynecologists)	ACOG	Subjects report 1 or more of the following affective and somatic symptoms at day −5 before menses in each of 3 prior menstrual cycles	
		Affective	Somatic
		Depression	Breast tenderness
		Angry outbursts	Abdominal bloating
		Irritability	Headache
		Anxiety	Swelling of extremities
		Confusion	
		Social withdrawal	
		Symptoms relieved within 4 days after menses onset without recurrence until at least cycle day 13. Symptoms present in absence of any pharmacologic therapy, hormone ingestion, or drug or alcohol use.	
		Symptoms occur reproducibly during two cycles of prospective recording	
		Subjects suffer from identifiable dysfunction in social or economic performance	
Premenstrual syndrome	PMS	PMS: subjects who scored ≥70 on the total Daily Record of Severity of Problems (DRSP) score during day 24–28 of menstrual cycle, and in addition there is a difference of at least 30% in DRSP scores between pre (late luteal phase day 24–28) and post (mid follicular day 6–10) menstrual phases	
Peri-menstrual syndrome	PeriMS	Sum DRSP day 1 + day 2 + day 24 to 28 ≥ 307 (0.666 percentile value)	
Menstrual cycle associated symptoms	MCAS	Sum of all DRSP scores from day 1 to day 28 ≥ 1,050 (0.666 percentile value)	

The DRSP and plasma hormone levels were measured at four different time points, namely day 7 (T1), day 14 (T2), day 21 (T3) and day 28 (T4) during the menstrual cycle in order to analyze changes in DRSP and sex hormone levels during the menstrual cycle. A normal menstrual cycle generally ranges between 26–35 days with a mean duration of 28 days. T1 DRSP values represent measurements of the mid-follicular phase when estrogen levels are rising. T2 represents mid-cycle values when there is a decline in estrogen levels and ovulation occurs. T3 values represent the mid-luteal phase when progesterone levels reach their peak values. T4 represents the end of the cycle when all hormones levels decline to their nadir (Owen, 1975; Mihm et al., 2011; Messinis et al., 2014).

### Assays

Fasting blood was sampled at 8.00 a.m. to assay plasma oestradiol and progesterone using an immunoassay for the quantitative determination of oestradiol and progesterone using Cobas^®^ 601. The methods to measure both sex hormones are described at great length in our previous published work (Roomruangwong et al., 2019a, b). The intra-assay CV value is 1.2% for oestradiol and 2.3% for progesterone.

#### Statistical Analysis

Analysis of contingency tables (χ^2^ test) was used to check associations between categorical variables, while analysis of variance (ANOVAs) was used to check differences in continuous variables between diagnostic groups. Generalized estimating equation (GEE) analysis, repeated measures, was used to check the effects of time, diagnosis and the interaction time X diagnosis on the sex hormone levels, while adjusting for age, age menarche, length cycle and duration of menses. GEE analyses, repeated measurements, were also used to examine the associations between the DRSP values over time (T1, T2, T3, and T4) and steady state hormone levels (an average value of the sex hormones over the cycle) and changes in hormonal levels from T1 to T4. In addition, we used a distributed lag model to predict the DRPS values over time (dependent variable) by the current and lagged (1 week) values of the sex hormones. We also introduce the Δ hormone values in the analysis, i.e., current value – lagged value 1 week earlier (Δprog_lag, thus denoting the changes in progesterone levels 1 week before blood sampling). Moreover, we also used Generalized Linear Model (GLM) analysis, repeated measurements, and computed effect sizes for time, time X diagnosis and diagnosis. Binary logistic regression analysis was used to delineate the most important predictors of the diagnosis (dependent variables) using the sex hormone levels as explanatory variables. Factor analysis was used to examine the factor structure of the DRSP data. The factorability of the factor analysis was assessed using the KMO index, while we also computed Bartlett’s test of sphericity. The number of factors was based on the number of factors with eigenvalues > 1. We performed equamax rotation of the relevant factors in order to interpret the factors and loadings ≥ 0.5 were considered to be significant. Tests were 2-tailed and a p-value of 0.05 was considered for statistical significance. All statistical analyses were performed using IBM SPSS windows version 25. The number of subjects was computed *a priori* (G^∗^Power 3.1.7): using a power of 0.8, effect size *f* = 0.22, two study groups and four measurements with a *r* = 0.4 correlation in a repeated measurement design (within–between interaction) showed that the total sample size should be 36. Therefore, we included 41 subjects divided into two groups.

## Results

### Different PMS Diagnoses

Table 1 lists the four PMS diagnoses used in the present study, two of these PMS diagnoses were already described in the section “Materials and Methods.” Based on the inspection of the daily values of the patients we decided to construct two new diagnoses, namely a first reflecting increased DRSP ratings in the peri-menstrual period (named: “PeriMS”) and a second showing increased ratings all over the menstrual cycle (named “menstrual cycle associated symptoms” or MCAS). The PeriMS index was computed as sum of all daily DRSP values at days 1, 2, 24, 25, 26, 27, and 28 ≥ 307, that is the 0.666 percentile of the distribution of the DRSP sums. The MCAS was computed as sum of all DRSP scores from day 1 to day 28 ≥ 1050, that is the 0.666 percentile of the DRSP sum distribution.

### Comparisons of the Effects of the Four Diagnoses on the DRSP Time Series

In order to evaluate the validity of the four diagnoses used in the current study, we have used GEE analysis with the time series of the total DRSP scores as dependent variables. Important predictors were the time effects (effects of time all over 28 days) and especially the diagnosis (four different diagnosis) X time interaction, namely the differences in the time series in participants with and without one of the four diagnoses. A greater impact of time X diagnosis is an important feature of a valid diagnosis, while also greater inter-group differences are important especially for the MCAS diagnosis. In addition, we have computed partial eta squared values using GLM repeated measurements analyses for time, time X diagnosis and group effects.

Table 2 shows the results of those GEE analyses. The ACOG diagnosis yielded significant time (partial eta squared η^2^ = 0.256) and less significant time X diagnosis (η^2^ = 0.094) effects, while the inter-group differences were not significant (η^2^ = 0.052). The PMS diagnosis yielded a significant time effect (η^2^ = 0.282), a significant time X diagnosis effects (η^2^ = 0.112) and showed a lower impact of inter-group differences (η^2^ = 0.150). The diagnosis PeriMS yielded significant time (η^2^ = 0.297) and time x diagnosis (η^2^ = 0.132) effects, while there were also highly significant inter-group differences (η^2^ = 0.689). The diagnosis MCAS yielded significant time (η^2^ = 0.312) and time x diagnosis (η^2^ = 0.103) effects, while the inter-group differences were highly important (η^2^ = 0.825).

**TABLE 2:** Results of GEE analysis with the daily total scores on the Daily Record of Severity of Problems (DRSP) as dependent variables and different PMS group, time and time X group interaction as independent variables.

**Group variables**	**Time**			**Group**			**Time X diagnosis**			**Daily mean (SE)**	
				
	***X*2**	**df**	***P***	***X*^2^**	**df**	***p***	***X*^2^**	**df**	***p***	**No**	**Yes**
ACOG	362.77	27	<0.001	2.58	1	0.108	187.64	27	<0.001	31.37 (1.77)	34.87 (1.45)
PMS	605.89	27	<0.001	5.33	1	0.021	103.95	27	<0.001	30.60 (1.37)	36.32 (1.64)
PeriMS	822.05	27	<0.001	81.05	1	<0.001	288.63	27	<0.001	27.72 (0.44)	40.00 (1.30)
MCAS	732.76	27	<0.001	180.56	1	<0.001	285.67	27	<0.001	28.58 (0.54)	42.71 (0.95)

*ACOG = Premenstrual syndrome diagnosis according to American College of Obstetricians and Gynecologists (ACOG) criteria. PMS = Subjects who scored ≥70 of total DRSP score during day 24–28 of menstrual cycle and there is a difference of at least 30% in DRSP scores between pre (late luteal phase day 24–28) and post (mid follicular day 6–10) menstrual phases. PeriMS = peri-menstrual syndrome. MCAS = menstrual cycle-associated symptoms.*

### Prediction of the Diagnostic Categories Using Hormonal Levels

Table 3 shows the results of binary regression analysis with the diagnosis as dependent variable and hormonal levels as explanatory variables. We also entered age, duration of menses, age menarche and length of the cycle, but these variables did not reach significance and therefore we omitted these extraneous variables from the final models shown in Table 3. We entered separately the four oestradiol values and the four progesterone data at T1, T2, T3, and T4 and based on these findings we constructed new z unit weighted composite scores, namely (a) sum of z scores of T2 progesterone (z T2 progesterone) + z T3 progesterone + z T4 progesterone (Prog T2 + T3 + T4); and (b) z T1 progesterone – z(Prog T2 + T3 + T4) Prog T1 –(T2 + T3 + T4). For oestradiol we found that two composite scores were useful, namely (a) sum of all z scores of the four oestradiol measurements (Oest T1 + T2 + T3 + T4) and (b) sum of the *z* scores at T2, T3, and T4 (Oest T2 + T3 + T4).

**TABLE 3:** Results of binary logistic regression analysis with different diagnoses as dependent variables.

**Dichotomies**	**Exploratory variables**	***X*^2^**	**Nagelkerke**	**Wald**	**df**	***p***	**OR**	**95% CI**
ACOG	–	–	–	–	–	–	–	–
PMS	Prog T1−(T2 + T3 + T4)	9.30	0.277	6.08	1	0.002	3.44	1.29–9.16
PeriMS	Prog T2 + T3 + T4	8.36	0.252	5.69	1	0.004	0.32	0.13–0.82
PeriMS	Oest T2 + T3 + T4	5.66	0.176	4.78	1	0.017	0.68	0.49–0.96
MCAS	Prog T2 + T3 + T4	9.44	0.289	6.55	1	0.002	0.31	0.13–0.76
MCAS	Oest T1 + T2 + T3 + T4	5.57	0.179	4.49	1	0.018	0.70	0.50–0.97

*ACOG = Premenstrual syndrome diagnosis according to American College of Obstetricians and Gynecologists (ACOG) criteria. PMS = Subjects who scored ≥70 of total DRSP score during day 24–28 of menstrual cycle and there is a difference of at least 30% in DRSP scores between pre (late luteal phase day 24–28) and post (mid follicular day 6–10) menstrual phases. PeriMS = peri-menstrual syndrome. MCAS = menstrual cycle-associated symptoms. OR: Odd’s ratio, 95% CI: 95% confidence intervals. Prog: progesterone; Oest: oestradiol. T1−(T2 + T3 + T4): computed as a z unit weighted composite score of zT1 – z(zT2 + zT3 + zT4). T1 + T2 + T3 + T4: sum of the z scores of the four time points. T2 + T3 + T4: sum of the z scores of the three time points in the luteal phase.*

Table 3 shows that the diagnosis according to ACOG criteria was not significantly predicted by any of the hormone levels or composite scores. PMS was significantly associated with the Prog T1 – (T2 + T3 + T4) score, but not with oestradiol levels. PeriMS was significantly predicted by Prog T2 + T3 + T4 and by Oest T2 + T3 + T4. The impact of the progesteron *z* score (Nagelkerke = 0.252) was greater than that of the oestradiol score (Nagelkerke = 0.176), and there were no cumulative effects of progesterone and oestradiol predicting PeriMS. We also found that MCAS was significantly predicted by Prog T2 + T3 + T4 or Oest T1 + T2 + T3 + T4.

### Features of PeriPMS

Table 4 shows the features of PeriMS versus no PeriMS. Thus, there were no significant differences in age, family income, age at menarche, length of the cycle, duration of menses, education, gave birth yes or no, and BMI between both study groups (results of ANOVAs or *X*^2^ tests). GEE analysis showed that the total DRSP score (sum of all 28 days) was significantly higher in subject with PeriMS than in those without (effect size: 0.689). The increases in the DRSP score in the pre- and post-menstrual weeks were significantly higher in women with than without PeriMS. Moreover, the impact of PeriMS on the DRSP ratings in the premenstrual week (η^2^ = 0.441) were more important than in the postmenstrual week (η^2^ = 0.217). The mean DRSP values averaged over T1, T2, T3, and T4 was significantly greater in women with PeriMS than in those without. This table shows also the measurements of oestradiol and progesterone at the four time points. We found that the levels of oestradiol at T2 and T3 and progesterone at T3 were significantly lower in women with PeriMS than in those without PeriMS.

**TABLE 4:** Measurement of Daily Record of Severity of Problems (DRSP) total score and subscales and demographic data in subjects with and without peri-menstrual syndrome (PeriMS).

**Variables**	**No PeriMS**	**PeriMS**	***F*/*X*^2^**	**df**	***p***	**Partial eta squared**
Age (years)	31.0 (6.6)	31.5 (7.7)	0.05	1/39	0.831	–
Family income (baht)	105.250 (104.245)	64.263 (42.911)	2.53	1/39	0.120	–
Age menarche (years)	12.7 (1.2)	12.9 (1.3)	0.30	1/39	0.588	–
Length cycle (days)	27.7 (2.4)	27.4 (5.7)	0.09	1/39	0.763	–
Duration menses (days)	4.4 (1.3)	5.0 (1.5)	2.17	1/39	0.149	–
Education (years)	16.0 (0.8)	15.9 (1.4)	0.05	1/39	0.818	–
Body mass index (kg/m2)	21.7 (3.5)	22.9 (3.8)	1.08	1/39	0.305	–
Children No/Yes	19/3	13/6	Ψ = 0.216	–	0.166	–
DRSP (28 days)	774.8 (58.0)	1119.6 (162.6)	86.53	1/39	<0.001	0.689
DRSP premenstrual week	132.6 (18.2)	252.2 (99.3)	30.82	1/39	<0.001	0.441
DRSP postmenstrual week	133.1 (19.9)	175.7 (56.9)	10.83	1/39	<0.001	0.217
DRSP (T1,T2,T3,T4)	27.0 (1.3)	38.3 (1.9)	35.56	1/157	<0.001	0.185
Oestradiol T1 (pmole/L)	266.1 (195.5)	315.3 (356.7)	0.019	1/38	0.892	–
Oestradial T2 (pmole/L)	723.9 (528.2)	476.5 (399.2)	4.50	1/38	0.040	–
Oestradiol T3 (pmole/L)	669.3 (260.1)	518.9 (292.7)	4.62	1/38	0.038	–
Oestradiol T4 (pmole/L)	308.9 (183.9)	252.1 (143.0)	0.85	1/38	0.361	–
Progesterone T1 (nmole/L)	0.51 (0.28)	0.56 (0.39)	1.45	1/38	0.236	–
Progesterone T2 (nmole/L)	4.33 (7.82)	2.68 (3.39)	0.26	1/38	0.614	
Progesterone T3 (nmole/L)	39.72 (23.43)	29.05 (30.16)	6.63	1/38	0.014	
Progesterone T4 (nmole/L)	13.71 (13.49)	8.95 (12.32)	2.54	1/38	0.120	
DRSP depression score	9.7 (0.5)	14.3 (0.6)	34.37	1/157	<0.001	0.180
DRSP physio-somatic score	6.8 (0.4)	10.1 (0.4)	36.77	1/157	<0.001	0.190
DSRP eating-breast score	5.0 (0.3)	6.6 (0.3)	13.42	1/157	<0.001	0.079
DRSP anxiety score	5.6 (0.3)	7.9 (0.3)	27.81	1/157	<0.001	0.150

*All results are shown as mean (SD); All hormonal data were processed in Ln transformation.*

### Results of Principal Component Analysis

Table 5 shows the results of PCA performed on the items of the DRPS in order to detect meaningful latent constructs that consequently could be used as severity indices of the underlying constructs. The analysis was performed on the 40 participants including the four time points (thus 160 cases). One item (worthlessness) did not load significantly on the PCs and showed less variation and therefore this item was removed from the final analysis. The factorability of the analysis was adequate (KMO = 0.888) and Bartlett’s test of sphericity was adequate (*X*^2^ = 3545.91, df = 253, *p* < 0.001). There were four factors with eigenvalues > 1 and explaining 73.11% of the variance. Table 2 shows the equamax rotated PCs: the first rotated PC explained 20.14% of the variance and loaded highly on depression, mood swings, sensitive to rejection, angry-irritable, more conflicts, less interest, out of control, and interference with hobbies and relationships. Therefore, we named this PC the “depressive dimension.” The second rotated PC explained 18.02% of the variance and loaded highly on concentration disturbances, lethargy, sleepiness, headache, muscle/joint pain and lowered productivity, and therefore we named this PC the “physio-somatic dimension.” The third rotated PC explained 17.83% of the variance and loaded highly on appetite and craving and breast tenderness and swelling, and therefore was named the “eating & breast PC.” The fourth rotated PC explained 17.11% of the variance and scored highly on hopelessness, anxious, lethargy, insomnia, being overwhelmed, and muscle-joint pain and was therefore named the “anxiety PC.” Consequently, we have computed the scores of the four different dimensions by adding up the symptoms belonging to the PCs and as such these sums reflect severity of the four underlying constructs of the DRSP.

**TABLE 5:** Results of factor analysis (equamax rotation) performed on the items of the Daily Record of Severity of Problems (DRSP) rating scale during 28 days of the menstrual cycle.

	**Component**			
	
	**1**	**2**	**3**	**4**
Depression	**0.563**	0.294	0.203	0.488
Hopelessness	0.333	−0.032	0.220	**0.746**
Anxious	0.469	0.234	0.267	**0.578**
Mood swings	**0.717**	0.278	0.158	0.371
Sensitive to rejection	**0.524**	0.291	0.374	0.485
Angry-irritability	**0.719**	0.336	0.222	0.240
More conflicts	**0.785**	0.236	0.223	0.159
Less interest	**0.577**	0.391	0.401	0.182
Concentration	0.307	**0.799**	0.008	0.173
Lethargy	0.122	**0.601**	0.209	**0.629**
Appetite	0.117	0.466	**0.619**	0.316
Craving	0.132	0.313	**0.716**	0.317
Sleepiness	0.215	**0.593**	0.363	0.313
Insomnia	0.066	0.072	0.154	**0.831**
Overwhelmed	0.478	0.364	0.262	**0.522**
Out of control	**0.613**	0.185	0.488	0.290
Breast tenderness	0.252	0.094	**0.772**	0.187
Breast swelling	0.206	0.156	**0.838**	0.183
Headache	0.240	**0.663**	0.349	0.060
Muscle/joint pain	0.096	**0.514**	0.207	**0.630**
Productivity	0.405	**0.705**	0.415	0.082
Hobbies	**0.538**	0.496	0.490	0.199
Relationships	**0.557**	0.448	0.489	0.177

*Extraction Method: Principal Component Analysis; Rotation Method: Equamax with Kaiser Normalization. Significant loadings are shown in bold (≥0.5). depression DRSP = Felt depressed, sad, “down,” or “blue” (item 1a). hopelessness DRSP = Felt hopeless (item 1b). Anxious = Felt anxious, tense, “keyed up” or “on edge” (item 2). Swing = Had mood swings (e.g., suddenly felt sad or tearful) (item 3a). Sensitive = Was more sensitive to rejection or my feelings were easily hurt (item 3b). Angry = Felt angry, irritable (item 4a). Conflict = Had conflicts or problems with people (item 4b). less interest = Had less interest in usual activities (e.g., work, school, friends, hobbies) (item 5). concentration = Had difficulty concentrating (item 6). Lethargy = Felt lethargic, tired, fatigued, or had a lack of energy (item 7). Appetite = Had increased appetite or overate (item 8a). Craving = Had cravings for specific foods (item 8b). Sleep = Slept more, took naps, found it hard to get up when intended (item 9a). Insomnia = Had trouble getting to sleep or staying asleep (item 9b). Overwhelm = Felt overwhelmed or that I could not cope (item 10a). out of control = Felt out of control (item 10b). tenderness = Had breast tenderness (item 11a). Swelling = Had breast swelling, felt “bloated”, or had weight gain (item 11b). Headache = Had headache (item 11c). muscle/joint pain = Had joint or muscle pain (item 11d). productivity = At work, at school, at home, or in daily routine, at least one of the problems noted above caused reduction of productivity or inefficiency. Hobbies = At least one of the problems noted above interfered with hobbies or social activities (e.g., avoid or do less). Relationships = At least one of the problems noted above interfered with relationships with others.*

### Measurements and Predictions of the Four DRSP Dimensions

Table 4 shows the measurements of these four dimensions in subjects with and without PeriMS. Thus, PeriMS was characterized by significantly higher scores of the four dimensions, with a strong impact on the physio-somatic (η^2^ = 0.190), depressive (η^2^ = 0.180) and anxiety (η^2^ = 0.150) dimensions and a lower effect size on the eating & breast dimension (η^2^ = 0.079).

Table 6 shows the results of GEE analyses, which examined the effects of oestradiol and progesterone time series as well as the hormone composite scores on the time series of the DSRP and its four dimensions. These analyses were performed in all 40 patients considering the four repeated measurements of the DPRS and hormones (denoted as T1→T4) or the composite scores of three or four time points (thus one fixed variable per subject). We found that the total DRSP score was significantly predicted by Prog (T1→T4) (inversely), but not oestradiol (T1→T4) or other variables. The sum DRPS (T1→T4) was also significantly predicted by the cumulative effects of changes over time in oestradiol (T1→T4) (inversely) and Prog T1−(T2 + T3 + T4) (positively). The same combination of variables also predicted the severity of the depressive and physio-somatic dimensions. The sum of the DRSP items and the depression and physio-somatic symptoms was also significantly predicted by the lagged progesterone (but not oestradiol) values. The total DRSP and depression scores were significantly predicted by progesterone T2 + T3 + T4 coupled with and Δprog_lag. The four repeated measurements of the anxiety and the eating-breast dimension were best predicted by Prog T2 + T3 + T4 (inversely) and Prog_lag, although the latter was also predicted by Oest T1 + T2 + T3 + T4 coupled with Pro_lag. The changes over time in progesterone (T1→T4) were predicted by oestradiol T1 + T2 + T3 + T4, Δoestradiol_lag and a time factor.

**TABLE 6:** Results of GEE analysis (repeated measurement) with the Daily Record of Severity of Problems (DRSP) total score and subscale scores as dependent variables.

**Dependent variables**	**Exploratory variables**	***B***	**SE**	**Wald *X*^2^**	**df**	***p***
Sum DRSP (T1→T4)	Oestradiol (T1→T4)	0.022	0.025	0.80	1	0.370
	Progesterone (T1→T4)	–0.076	0.023	10.79	1	0.001
Sum DRSP (T1→T4)	Oestradiol (T1→T4)	–0.059	0.022	6.92	1	0.009
	Progesterone T1−(T2 + T3 + T4)	0.082	0.023	12.80	1	<0.001
Sum DRSP (T1→T4)	Prog_lag	0.084	0.039	4.76	1	0.029
Sum DRSP (T1→T4)	ΔProg_lag	–0.075	0.025	9.34	1	0.002
Sum DRSP (T1→T4)	Progesteron T2 + T3 + T4	–0.116	0.020	35.11	1	<0.001
	Prog_lag	0.118	0.037	10.49	1	0.001
Sum DRSP (T1→T4)	Progesteron T2 + T3 + T4	–0.081	0.023	12.52	1	<0.001
	ΔProg_lag	–0.074	0.025	8.87	1	0.003
Depression (T1→T4)	Oestradiol (T1→T4)	–0.059	0.021	7.74	1	0.005
	Progesterone T1−(T2 + T3 + T4)	0.096	0.022	20.01	1	<0.001
Depression (T1→T4)	Progesterone T1−(T2 + T3 + T4)	1.423	0.368	14.96	1	<0.001
	ΔProg_lag	–0.048	0.019	6.05	1	0.014
Physio-somatic (T1→T4)	Oestradiol (T1→T4)	–0.061	0.030	4.02	1	0.045
	Progesterone T1−(T2 + T3 + T4)	0.076	0.030	6.25	1	0.012
Physio-somatic (T1→T4)	Progesterone T2 + T3 + T4	–0.971	0.299	10.50	1	0.001
	Prog_lag	1.410	0.574	6.03	1	0.014
Anxiety (T1→T4)	Progesterone T2 + T3 + T4	–0.979	0.365	7.22	1	0.007
	Prog_lag	0.929	0.404	5.28	1	0.022
Eating-& Breast (T1→T4)	Progesterone T2 + T3 + T4	–0.517	0.131	15.50	1	<0.001
	Prog_lag	1.09	0.423	6.60	1	0.010
Eating-& Breast (T1→T4)	Oestradiol (T1 + T2 + T3 + T4)	–0.211	0.072	8.54	1	0.003
	Prog_lag	1.023	0.420	5.92	1	0.015
Progesterone (T1→T4)	Oestradial (T1 + T2 + T3 + T4)	0.167	0.040	17.74	1	<0.001
	Δoestradiol_lag	–0.383	0.101	14.26	1	<0.001
	Time	1.478	0.354	187.79	1	<0.001

*T1→T4: denotes the repeated measurements over four time points. T1−(T2 + T3 + T4): computed as a z unit weighted composite score. T1 + T2 + T3 + T4: sum of the z scores of the four time points. T2 + T3 + T4: sum of the z scores of the three time points in the luteal phase. Prog_log: lagged progesterone values (1 week lag). ΔProg_lag and Δoestradiol_lag: delta values of current hormonal values – lagged hormonal values (1 week lag).*

## Discussion

The first major finding of the current study is that lowered steady state levels of sex hormones, mainly progesterone and to a lesser degree also oestradiol, when averaged over the menstrual cycle, predicted the presence of PMS as well as its severity. Previous studies that assessed associations between progesterone levels and the onset of PMS have yielded controversial results. For example, in women with PMDD peripheral levels of progesterone or its active metabolite allopregnanolone during the luteal phase were found to be either decreased (Wang et al., 1996; Rapkin et al., 1997; Ziomkiewicz et al., 2012) or increased (Backström et al., 1983; Hammarbäck et al., 1989; Girdler et al., 2001) across different studies, whilst some other studies found no significant changes in these hormones (Rubinow et al., 1988; Hsiao et al., 2004).

Discrepancies in some of the above-mentioned case-control studies and the current study may be explained by our findings that the severity of PMS symptoms (which as a scale variable is a more sensitive score of PMS symptoms than a categorical PMS diagnosis) is predicted by steady state levels of progesterone combined with changes over time in progesterone levels in a distributed lag model (with current and lagged values of the sex hormones). For example, changes in DRSP scores from the start of the cycle until the end of the luteal phase were significantly associated with lowered steady levels of progesterone combined with lagged changes in progesterone levels, indicating that when plasma levels of progesterone decline in the luteal phase (the week prior to DRSP measurements) severity of PMS is worse. These findings contrast with previous results that found the onset of PMDD to be associated with fluctuations in oestradiol and progesterone levels during the menstrual cycle, but not with their steady-state levels (Schmidt et al., 2017). Moreover, blocking the conversion of progesterone to its metabolite using the 5α-reductase inhibitor dutasteride mitigates symptoms of PMDD (Martinez et al., 2016). A recent study, which examined salivary progesterone, found that in women with PMS, progesterone levels declined rapidly 3 days prior to menstruation whereas mid-cycle progesterone concentrations were similar to those of asymptomatic participants (Lovick et al., 2017). Similar results were also reported by Andréen et al. (2006) who reported that participants who developed PMS symptoms had an increased severity score during the period when progesterone was stable and then further increased when progesterone levels declined rapidly by the end of the cycle. Animal studies also demonstrate reproducible depressive-like behaviors during various progesterone withdrawal protocols (Li et al., 2012).

Moreover, we found that lowered steady state levels of progesterone averaged over the cycle coupled with changes over time in oestradiol levels showed that the latter had a significant effect on DRSP scores, although this effect disappeared when progesterone changes over time were taken into account. This indicates that putative detrimental effects of oestradiol could at least in part be mediated (statistically) by progesterone levels. These results suggest that PMS is related to lower progesterone concentrations during the second half of the menstrual cycle, which is described as “corpus luteum insufficiency,” and is considered to result from suboptimal pre-ovulatory follicular development (Dawood, 1994; Hinney et al., 1996). Deficient progesterone production is a condition that may lead to inadequate maintenance of a regular secretory endometrium and, therefore, may be associated with recurrent pregnancy loss and infertility, although up to 10% of fertile women also show corpus luteum insufficiency (Sonntag and Ludwig, 2012). This condition may be due to a functional inadequacy of the hypothalamic-pituitary secretion of gonadotrophins, or may otherwise occur in patients suffering from the polycystic ovary syndrome (PCOS) (Barthelmess and Naz, 2014). Whereas the former may be related to external factors, including exposure to environmental xeno-oestrogens, the latter is commonly associated with insulin resistance and metabolic disturbance.

The second major finding of this study is that the diagnostic criteria used to establish a diagnosis of PMS may determine to a large extent outcomes of biomarker studies. In this respect, we found that the diagnoses PMS, PeriMS and MCAS were externally validated by levels of sex hormones, whereas ACOG-based diagnosis of PMS was not associated with peripheral levels of sex hormones. Furthermore, the ACOG diagnosis performed less robustly in GEE analyses examining the effects of time and diagnosis on DRSP scores. It is interesting to note that both psychiatrists (PMS diagnosis) and gynecologists (ACOG) have developed overlapping but distinct sets of criteria for PMS. It seems clear, however, that a diagnosis of PMS based on ACOG criteria may not reflect the severity of PMS symptoms premenstrually, but merely screens a few symptoms premenstrually. In addition, we found that the diagnosis PMS was only predicted by steady state levels of progesterone, while the PeriMS and MCAS diagnoses were significantly related to both sex hormones. Furthermore, the PMS diagnosis may be less adequate because all DRSP scores during the cycle are significantly intercorrelated and thus using a 30% difference between the premenstrual and postmenstrual phases may fail to identify some “true” PMS cases, namely those who have high scores all over the menstrual cycle in addition to PMS.

Therefore, we have developed two new diagnoses based on DRSP scores during the cycle: (a) the peri-menstrual syndrome (PeriMS), which considered total DRSP scores on day 1 + day 2 + day 24 to 28 with a cut-off score at the 0.666 percentile to dichotomize the peri-menstrual DRSP score; and (b) menstrual-cycle associated symptoms (MCAS, using a cut-off score at the 0.666 percentile), which delineates a group of subjects with increased DRSP levels during the cycle. Our results show that changes in sex hormones during the menstrual cycle and lowered steady state levels of these hormones determine increased peri-menstrual symptoms and increased ratings during the cycle, rather than “premenstrual” symptoms.

The third major finding of this study is that using factor analysis we were able to detect four interpretable factors in the DRSP data set, namely (a) a depressive dimension; (b) a physio-somatic component (with symptoms reminiscent of chronic fatigue); (c) increased appetite and craving combined with breast tenderness and swelling; and (d) an anxiety dimension. A previous factor analysis study also yielded four factors, namely (a) mood symptoms (depressed/sad/blue, mood swings, angry/irritability, anxious/tension/on edge, overwhelmed, sensitive to rejection, worthless/guilty, out of control, hopeless, conflicts/problems, less interest and trouble sleeping); (b) behavioral symptoms (lethargy/tired/fatigue, difficulty concentrating, sleepiness, craving specific foods, and increased appetite); (c) pain symptoms domain (two items including headache and joint/muscle pain); and (d) physical symptoms (four items including breast tenderness and breast swelling/bloating) (Wu et al., 2013). Thus, both factor analysis studies suggest the presence of at least three different dimensions, namely an affective, a behavioral (or physio-somatic) and a breast swelling dimension. Most importantly, we found that the peri-menstrual syndrome was characterized by increased scores on all four dimensions and that changes during the cycle in severity of those dimensions were significantly associated with steady state levels and (lagged) changes in sex hormones, mainly progesterone. This suggests that the four symptom dimesions measured with the DRSP are in part mediated by sex hormones.

An important question is how these sex hormones could exert their effect on the different DRSP dimensions. Progesterone receptors can be found throughout human brain including the caudate, hippocampus, hypothalamus and limbic system (Maggi and Perez, 1985). The limbic system, which modulates emotion and behavior, is influenced by circulating progesterone. For example, progesterone metabolites have antagonistic properties at GABA-A receptors and increase the metabolism and turnover of monoamines in the brain, which may lead to negative mood, including anxiety and depression (Panay and Studd, 1997). Changes in progesterone or its metabolites may induce GABA-A receptor dysfunctions that may increase susceptibility to develop PMS (Timby et al., 2016) and changes in estrogens or progesterone during the luteal phase may cause changes in dopamine receptors sensitivity (Wieck et al., 2003; Czoty et al., 2009; Seeman, 2012). Moreover, estrogen may impact depressive symptoms. For example, women with severe PMS show clinical improvements when cycles were absent during pregnancy to recur after birth as postnatal depression when estrogen levels fall (Studd, 2015). Previous functional magnetic resonance imaging studies during different menstrual phases showed effects of estrogen in attenuating arousal pathways in women and modulating the stress response (Goldstein et al., 2005, 2010).

Estrogen may inhibit food intake, whereas progesterone stimulates appetite (Hirschberg, 2012). Interestingly, some studies found low mean food intake during the mid-cycle of the menstrual cycle when estradiol levels are high, whereas the peak food intake occurs during the premenstrual period when progesterone levels are high (Buffenstein et al., 1995; Reed et al., 2008). Therefore, lower levels of estrogen in the luteal phase could explain increased appetite and craving in individuals with PMS. In addition, many physical symptoms associated with progesterone, including edema, weight gain, bloating, and breast tenderness, may be related to its mineralocorticoid-like effects, which enhance the renin–aldosterone cascade (Oelkers et al., 1974). Therefore, progesterone may compete for the mineralocorticoid receptor, leading to fluid and sodium retention during the luteal phase (Panay and Studd, 1997).

The classic-school allopathic approach to treatment of patients suffering from PMS recommends suppressing ovulation by a combined oral contraceptive. However, this approach does commonly not relief the symptoms, which is part is related to the type of progestagen used, with drospirenone possibly being preferable (Nevatte et al., 2013). For example, in a subgroup of patients, hormonal contraceptive pills, despite of suppressing the ovulation, may increase PMS-like symptoms (e.g., irritability, depression, anxiety, bloating, fatigue, and breast tenderness) (Oinonen and Mazmanian, 2002). Overall, progesterone treatment studies did not reveal an efficacy of progesterone to treat PMS or PMDD (Freeman et al., 1990; Ford et al., 2012). In addition, more than half of women who started taking hormonal contraceptives discontinue these drugs within the first year due to side effects including PMS-like symptoms (Berenson et al., 1997; Rosenberg and Waugh, 1998; Doyle et al., 2007). Other studies showed that cyclical and continuous progestogen treatment may induce PMS-like symptoms (Baker and O’Brien, 2012). Women with PMS may experience more PMS-like symptoms after administration of a gonadotropin-releasing hormone analog (GnRHa) followed by exogenous estrogen or progesterone administration (Schmidt et al., 1998).

Future research on the treatment of PMS should trial Clomiphene citrate (given the first 5 days of the cycle) and a mid-cycle injection of human Chorionic Gonadotrophin in subjects with peri-menstrual syndrome. Clomiphene citrate is an anti-oestrogen with complementary intrinsic oestrogenic activity, which is the treatment of choice for suboptimal follicular development. This medication should be given during the first 5 days of the cycle, and may be combined with a mid-cycle injection of human Chorionic Gonadotrophin (hCG), which increases endogenous progesterone secretion during the luteal phase. Nevertheless, some patients may experience adverse effects of Clomiphene and may prefer to combine injections of human Menopausal Gonadotrophin (hMG) on days 8 and 12 of the cycle, with a mid-cycle hCG injection. Anecdotal case studies using this approach have been published, but well-designed clinical trials are lacking so far. Hyperinsulinism and metabolic disturbance due to insulin resistance in PCOS patients can be treated with Metformin, but the Ayurvedic plant extract of *Momordica charantia* (bitter gourd) may be preferable because of its more favorable toxicological profile (Comhaire, 2014).

The results of the current study should be interpreted within its limitations. First, we enrolled a small sample (*n* = 41), although the power of the GEE, repeated measurements, analyses was adequate (>0.8). Second, it would have been more interesting if we had sampled both sex hormones on a daily basis to be able to perform group spectral analyses to examine associations between the cycles in DRSP ratings and hormones in the different study groups (Maes et al., 1995).

## Conclusion

In conclusion, the cumulative effects of lowered steady state levels of progesterone in the luteal phase combined with (lagged) changes in progesterone in the luteal phase predict total DRSP scores as well as its four main dimensions (namely depression, physio-somatic symptoms, breast tenderness and appetite, anxiety) and, therefore, the diagnosis of peri-menstrual syndrome. Classical diagnoses of PMS are less adequate, whereas two new diagnoses developed in the current study are externally validated by the biomarkers, namely (a) a diagnosis of peri-menstrual syndrome denoting individuals with increased symptoms in the pre and post-menstrual period; and (b) a diagnosis of menstrual cycle-associated symptoms (MCAS) denoting subjects who experience increased DRSP symptoms all over the cycle. Therefore, future research should examine the associations of biomarkers with those two diagnoses and with changes over time in the DRSP (and its four dimensions), which provides more information on the steady state (increased scores all over the cycle) and cyclical nature (peri-menstrual) of the syndromes.

## Data Availability Statement

The datasets generated for this study will be made publicly available once the data set has been fully exploited by the authors. Requests to access the datasets should be directed to the corresponding author.

## Ethics Statement

The studies involving human participants were reviewed and approved by the Ethics Committee of the Faculty of Medicine, Chulalongkorn University, Bangkok, Thailand (IRB No.611/60, COA No. 1111/2017). The patients/participants provided their written informed consent to participate in this study.

## Author Contributions

CR and MM designed the study. CR recruited and screened the participants. MM performed the statistical analyses. AC and FC contributed in a meaningful way to the intellectual content of this manuscript. All authors agreed upon the final version of the manuscript.

## Conflict of Interest

The authors declare that the research was conducted in the absence of any commercial or financial relationships that could be construed as a potential conflict of interest.
